# Advancements in the Management of Severe Community-Acquired Pneumonia: A Comprehensive Narrative Review

**DOI:** 10.7759/cureus.46893

**Published:** 2023-10-12

**Authors:** Don Davis, Jainisha Thadhani, Vatsalya Choudhary, Reem Nausheem, Cristhian R Vallejo-Zambrano, Bushra Mohammad Arifuddin, Mujahaith Ali, Bryan J Carson, Fnu Kanwal, Lavanya Nagarajan

**Affiliations:** 1 Medicine, Medical University of Varna, Varna, BGR; 2 Medicine, Royal College of Surgeons in Ireland, Medical University of Bahrain, Manama, BHR; 3 Medicine, Kasturba Medical College of Manipal, Manipal, IND; 4 Medicine, Jilin Medical University, Jilin, CHN; 5 General Physician, Colegio de Médicos de Manabí, Manta, ECU; 6 Internal Medicine, Shadan Institute of Medical Sciences and Teaching Center, Hyderabad, IND; 7 Medicine, Ternopil National Medical University, Ternopil, UKR; 8 Emergency Medicine, Northern Health and Social Care Trust, Coleraine, GBR; 9 Medical College, Chandka Medical College, Larkana, PAK; 10 Department of Medicine, The Tamilnadu Dr.M.G.R. Medical University, Chennai, IND

**Keywords:** community-aquired pneumonia, guideline directed medical therapy, narrative review, severe community-acquired pneumonia, antibiotics therapy

## Abstract

Pneumonia, classified as a lower respiratory tract illness, affects different parts of the bronchial system as well as alveoli and can present with varying severities depending on co-morbidities and causative pathogens. It can be broadly classified using the setting in which it was acquired, namely the community or hospital setting, the former being more common and spreading through person-to-person droplet transmission. Community-acquired pneumonia (CAP) is currently the fourth leading cause of death worldwide, and its high mortality makes continual insight into the management of the condition worthwhile.

This review explores the literature specifically for severe CAP (sCAP) and delves into the diagnosis, various modalities of treatment, and management of the condition. This condition can be defined as pneumonia requiring mechanical ventilation in the ICU and/or presenting with sepsis and organ failure due to pneumonia. The disease process is characterized by inflammation of the lung parenchyma, initiated by a combination of pathogens and lowered local defenses. Acute diagnosis of the condition is vital in reducing negative patient outcomes, namely through clinical presentation, blood/sputum cultures, imaging modalities such as computed tomography scan, and inflammatory markers, identifying common causative pathogens such as *Streptococcus pneumoniae*, rhinovirus, *Legionella*, and viral influenza. Pathogens such as *Escherichia coli* **should also be investigated in patients with chronic obstructive pulmonary disease.

The mainstay of treating sCAP includes rapid ICU admission once a diagnosis has been confirmed, initiating sepsis protocol, and treatment with combined empiric antibiotic regimens consisting of beta-lactams and macrolides. Corticosteroid use alongside antibiotics shows promise in reducing inflammation, but its use has to be judged on a case-by-case basis. New drugs such as omadacycline, delafloxacin, and zabofloxacin have shown valid evidence for the treatment of resistant causative organisms. The main guidelines for preventing sCAP include maintaining a healthy lifestyle, and annual pneumococcal and influenza vaccines are recommended for the most vulnerable patient groups, such as those with COPD and immunosuppression.

## Introduction and background

Pneumonia is a typical acute respiratory illness affecting the distal bronchial tree and alveoli of the lungs. This condition can often be categorized as either community-acquired pneumonia (CAP) or hospital-acquired pneumonia (HAP), which includes ventilation-associated pneumonia (VAP). The microbiology of CAP and HAP differs depending on host risk factors, such as aberrant stomach and oropharyngeal colonization, and whether pneumonia is contracted in the community or a medical setting [1]. According to data from the 2019 Global Burden of Diseases study, approximately 489 million individuals globally were affected by lower respiratory tract infections, including pneumonia and bronchiolitis. The study also revealed that pneumonia predominantly impacts individuals under the age of 5 and those over the age of 70 [1].

CAP is the leading infectious cause of mortality and ranks as the fourth highest contributor to global deaths [2]. It is defined as pneumonia contracted outside the hospital in individuals who were not admitted to the hospital the month before the symptoms appeared [2]. The primary causative agents of CAP consist of *Streptococcus pneumoniae*, respiratory viruses, *Haemophilus influenzae*, and other bacterial species including *Mycoplasma pneumoniae* and *Legionella pneumophila*. The current criteria used to define severe CAP (sCAP) primarily focused on identifying patients who present with septic shock accompanied by organ failure and/or severe acute respiratory distress that necessitates invasive mechanical ventilation [3,4]. Even with the administration of effective antibiotic treatment, sCAP is linked to a significant mortality rate, ranging from 16% to 36%, often resulting in rapid patient demise [3,5].

Pharmacotherapy is commonly employed in the management of sCAP, with antibiotics serving as the primary therapeutic agents for bacterial pneumonia, and they should be initiated promptly after diagnosis. The concurrent administration of beta-lactam antibiotics like cefotaxime, in conjunction with macrolides, has shown improved rates of survival when compared to the use of beta-lactam monotherapy alone [6,7]. Steroids have been added to existing antibiotic regimens to treat some cases of sCAPs [8]. This helps target the inflammatory processes of the disease and improve prognosis. Evidence also supports the use of oseltamivir in treating severe pneumonia caused by influenza virus within the first 48 hours of diagnosis. Among the newer drugs, lefamulin, delafloxacin, and omadacycline have shown significant promise in treating sCAP [8-10].

This narrative review intends to explore our current understanding of SCAP management and summarize recent advancements in treatment options intended to enhance patient outcomes.

## Review

Epidemiology of sCAP

Every year, approximately 4 million adults in the United States are diagnosed with CAP [11]. According to one estimate, about 915,900 events of CAP occur annually in the US, with male patients and adults over 65 years of age more likely to be associated with severe complications [4]. Patients with severe CAP often experience refractory shock, refractory hypoxemia, and other pneumonia-related complications, including organ system failure, which are the leading causes of death [12].

Microbiology and etiology of sCAP

Bacterial infection, which is a component of the complex causes of sCAP, was the main concern in 1990 [13]. Currently, as suggested by bacteriologists around the world, there are several causes of sCAP, including viral infections, that can be identified by amplification of nucleic acid techniques [13]. In a Chinese study conducted on 275 patients hospitalized in 17 hospitals of sCAP between June 2018 and December 2019, it was discovered that the main pathogens were *S. pneumoniae*, *Enterobacteriaceae*, *L. pneumophila*, influenza virus, and *M. pneumoniae*. However, they also advised being cautious of uncommon bacteria, such as *Leptospira* and *Chlamydia psittaci* [14].

A Korean study included 198 patients who were being treated at the Asan Medical Center and Referral Hospital in Seoul, South Korea [15]. The researchers obtained samples of pathogens through blood and sputum cultures and performing bronchoalveolar lavage for Gram staining. This study showed that viral infection increased the chance of developing bacterial infections and was strongly linked to sCAP. The virus most commonly associated with sCAP is rhinovirus [15]. In a study conducted at European hospitals, which included 1576 sCAP patients between March 2016 and May 2020, it was demonstrated that 568 patients had SARS-CoV-2, 482 belonged to the influenza group, and 526 patients were not related to viral infections [16]. SARS-CoV-2 vaccinations now shield against the majority of serious illnesses [17].

In conclusion, the initial step in treating sCAP is to identify its cause. Laboratory methods, including blood cultures, sputum, BAL for Gram staining, and amplification of nucleic acids, can be used to detect pathogens [13,14]. According to these data, *S. pneumoniae*, *L. pneumophila*, *Enterobacteriaceae*, and *M. pneumoniae* are the principal bacterial pathogens connected to sCAP, as well as viral infections such as the influenza virus, rhinovirus, and SARS-CoV-2 among unvaccinated individuals [17].

Pathophysiology

Inflammation of lung tissue triggered by infectious microorganisms or changes in the alveolar microbiome [18], combined with weakened local defense mechanisms followed by the formation of inflammatory exudates in the alveoli [19], summarizes the pathogenesis of CAP. CAP can be initiated through various modes of infection [20]. Firstly, person-to-person transmission occurs through the inhalation of aerosolized droplets released by an infected individual. Particles as small as 5 μm can carry up to 100 microorganisms, evading respiratory defenses and reaching the alveoli. This is the primary route for healthy young individuals during community infections [19]. Secondly, microaspiration involves tiny particles and microorganisms from the upper airways entering the lower airways through aspiration. Conditions such as a weak cough reflex, altered consciousness, and impaired mucociliary mechanism increase the risk of microaspiration [21]. Macroaspiration and hematogenous spread also play roles in diverse modes of pneumonia infection. These mechanisms collectively contribute to the complex landscape of CAP transmission.

External stressors, such as smoking or viral infection, alter the native microbiome of alveoli, causing these microbes to replicate, which leads to the failure of alveolar macrophages to destroy them from infecting the alveoli, thereby initiating an inflammatory chemotactic response [22]. The lung defense system counters inhaled pathogenic bacteria by using mucus, cilia, and surfactant proteins. Alveolar macrophages clear bacteria, initiate immune responses, and release cytokines, such as TNF and IL-1, to attract phagocytic cells. Epithelial cells release antimicrobial peptides, whereas alveolar macrophages produce interferons and cytokines, which recruit neutrophils and monocytes. This influx of phagocytes aids in bacterial control, and the overall cytokine response remains consistent [23]. Immune resistance mechanisms include direct actions, such as neutrophil-produced hypochlorite, and coordinated efforts, such as chemokine-driven neutrophil recruitment. Tissue resilience endures microbial stress and reduces damage through antiproteases and cytokines [24]. Locally produced chemical molecules, such as interleukins, monocyte chemotactic protein-1, and granulocyte colony-stimulating factor, contribute to lung defense, triggering systemic inflammatory responses upon entering the bloodstream [22].

Pathogen Invasion

Bacteria release pore-forming toxins that cause cytolysis and disruption of host signaling. *S. pneumoniae*’s pneumolysin (PLY) forms pores in cholesterol-containing membranes, affecting immune cells and inducing cytokines [25]. *Staphylococcus aureus* forms microaggregates that interact with the alveolar epithelium, inducing alpha hemolysis-mediated damage [26]. Pore-forming toxins trigger cell death via inflammatory necroptosis independent of caspase activation. PLY also induces non-inflammatory apoptosis, which involves cellular contents within the membranes [25].

Role of Viral Infections in Compromising Pulmonary Immunity Against Bacterial Infections

Viral infections prime the lungs for bacterial superinfections and create vulnerable adherence sites. Bacterial superinfection takes advantage of this environment, often weakening the immune response against bacterial invasion [27]. Antimicrobial peptides and neutrophil recruitment are inhibited. Although neutrophils are recruited, they exhibit reduced bactericidal capacity, contributing to the immunopathology of sCAP [27,28]. IL-1 family cytokines [29] play a dual role, aiding bacterial infection while assisting host defenses during viral superinfection. Maintaining a balance between antibacterial immunity and inflammation is essential for favorable outcomes during superinfection. Understanding these dynamics will inform clinical strategies for the treatment of CAP.

Diagnosing sCAP

In the context of outpatient care, the combination of routine vital sign assessments and ordinary examination of the lungs indicates a low likelihood of CAP occurrence [30]. Additionally, various studies focusing on patient history assessment and physical examination have demonstrated that while certain factors may appear pertinent when dealing with suspected pneumonia cases, such as dullness to percussion, wheezing, and crackles, no singular observation or combination of observations can definitively confirm or dismiss the diagnosis [31,32]. Thus, to achieve a conclusive diagnosis, it is recommended to conduct imaging investigations, such as chest X-ray (CXR), CT scans, or lung ultrasound, in addition to blood tests, to determine if the criteria for sCAP (as outlined by the American Thoracic Society [33]) are satisfied.

Despite exhibiting less than perfect sensitivity and yielding inconsistent interobserver agreement [34], CXR (posteroanterior and lateral views) remains an indispensable modality for diagnosing CAP, owing to its cost-effectiveness and safety. Nevertheless, the utility of a negative outcome proves suboptimal in effectively excluding the disease; thus, chest CT or empirical treatment should be considered, given the potential for false-negative results in the initial CXR assessment [35,36].

The enhanced accuracy of chest CT in the diagnosis and management of CAP has demonstrated promising outcomes. Early CT scans prompted alterations in antimicrobial treatments and site-of-care for 60.8% of the patients, resulting in more tailored patient care and significantly boosting practitioner confidence in CAP diagnosis [37]. Subsequent investigations have advocated for the prioritization of CT scans as the primary imaging modality for specific individuals with suspected CAP. This strategy not only heightens diagnostic precision but also results in a significant net reclassification improvement, ranging from 8% to 18% among patients, thereby reducing the occurrence of unnecessary antibiotic prescriptions [38].

Guidelines for diagnosing CAP using microbiological tests have been modified because of the increasing prevalence of antibiotic-resistant pathogens. Numerous extensive observational studies have documented reduced mortality rates associated with the acquisition of blood cultures, sputum gram stains, and urine tests for pneumococcal and *Legionella* antigens from sCAP patients upon admission [33,39]. Additionally, for adults afflicted with CAP, the American Thoracic Society recommends the examination of influenza viruses at the point of diagnosis utilizing a rapid influenza molecular assay, owing to the significant advantages offered by antiviral therapy. However, this recommendation is applicable only when influenza is highly prevalent in a community setting [33].

Inflammatory biomarkers such as procalcitonin (PCT) and C-reactive protein (CRP) also play an important role in diagnosing sCAP. In healthy adults, CRP concentrations below 5 mg/L and PCT levels under 0.1 ng/mL are considered normal [40,41]. One study concluded that high CRP values are especially prevalent in pneumonia caused by *S. pneumoniae* or *L. pneumophila* [42]. Another study by Stolz et al. demonstrated that employing a CRP cut-off value of 100 mg/L resulted in a 91.2% specificity for predicting pneumonia [43,44]. Nevertheless, it is essential to emphasize that CRP is not a specific biomarker exclusively indicative of bacterial infection, as it can also manifest elevated levels in other conditions, including collagen vascular diseases [45,46]. In regard to PCT, studies suggest that it holds prognostic relevance for mortality in CAP, although its predictive value may be less pronounced than that of other biomarkers due to potential false results from conditions such as subacute endocarditis or renal failure [44]. Given the aforementioned considerations, it is not advisable to rely solely on CRP or PCT levels to determine the necessity of antibiotic treatment [47].

In conjunction with clinical judgment and other diagnostic techniques, the American Journal of Respiratory & Critical Care Medicine supports the use of the Pneumonia Severity Index (PSI) as an established clinical prognostic tool for assessing the need for hospitalization in adults with CAP as opposed to CURB-65 (a tool based on confusion, urea level, respiratory rate, blood pressure, and age ≥65 years) [33]. This preference stems from PSI’s ability to identify a broader range of patients classified as low-risk and its enhanced predictive power for mortality [48]. Substantial supportive evidence drawn from three clinical trials and one observational study further substantiates the efficacy and safety of PSI employment for sCAP [49-52]. However, caution is warranted as PSI could potentially underestimate the severity among younger patients and oversimplify the manner in which clinicians interpret continuous variables such as blood pressure. For instance, any systolic blood pressure <90 mmHg is considered anomalous irrespective of the individual’s baseline and observed readings [33].

Management options for community-acquired pneumonia

Exploring non-pharmacological management

Noninvasive Ventilation

Noninvasive ventilation (NIV) has emerged as an effective and viable option for managing CAP, in addition to the use of antibiotics. This approach involves providing ventilatory support to patients with acute respiratory failure (ARF) using devices such as nasal masks or helmets, which deliver positive pressure to the airways. The standard oxygen therapy and NIV are being used in patients with sCAP. High-flow nasal oxygen (HFNO) can also be used for severe CAP. Mechanical ventilation is used in patients showing signs of respiratory failure. According to an RCT [53], HFNO was associated with a reduction in intubation rates in patients with a PaO2/FiO2 ratio <200 mmHg with CAP. New data support helmet NIV to improve respiratory support, especially during strenuous exercise and severely depleted oxygenation (PaO2/FiO2 ratio < 100). Frat et al. [54] compared HFNO with conventional oxygen therapy. They studied 106 patients on HFNO, 110 on NIV, and 94 on standard oxygen therapy for ARF. HFNO did not significantly reduce intubation time, and ICU mortality was similar, but HFNO had lower in-hospital mortality and 90-day mortality compared with standard oxygen. The effects of HFNO were most pronounced during hospitalization, after which survival was affected by other factors.

Immunoglobulins

A variety of immunoglobulins play an important role in the protection against various infections. One of these is IgM, which is the most important and first to appear in the primary antibody response and protects against various bacterial infections. It is superior to IgG for rapid complement activation and bacterial opsonization [55]. A study by de al Torre et al. [56] included 98 patients who required ICU admission and demonstrated an increase in mortality due to a reduction in IgG levels. However, decreased levels of IgG, IgG1, and IgG2 were associated with an increase in 30-day mortality [57]. Various polyclonal antibodies, such as IVIG and IgM, have shown efficacy in the early stages of sCAP with sepsis [58,59]. IVIG developed for patients with sepsis and sCAP containing pentaglobin (12% IgM, 12% IgA, and 76% IgG) showed antibacterial and anti-inflammatory effects. Additionally, a CIGMA [60] study demonstrated the efficacy of trimodulin, a polyclonal antibody. Interestingly, IgM levels increased to 23%. Trimodulin antibody was more effective in patients with low IgM and high CRP levels.

Pharmacological treatment for sCAP

Pharmacological treatment of sCAP typically involves the use of antibiotics. Antibiotics are the mainstay of treatment for bacterial pneumonia and should be initiated promptly after diagnosis. It is also vital to initiate protocols early to ensure that sCAP patients receive early organ support, which could include admission to the intensive care unit (ICU). As detailed in a cohort study [61], patients with prolonged emergency room stay demonstrated not only a higher incidence of mortality but also a higher risk of developing sepsis once transferred to the ICU (OR = 2.5; 95% CI, 1.3-4.7).

Antimicrobial Regimen

The avid progression of sCAP into a profile demonstrates that of sepsis, with over half of the patients who are diagnosed with the condition requiring a hospital stay, in addition to mortality rates reaching 48% within seven days of being diagnosed [62], acute and accurate management of the condition is vital. Rapid administration of empiric antibiotic combinations has been shown to decrease the mortality rate of patients [63-65]. Routinely, when treating bacterial pneumonia, it is best to cover all the common causative pathogens, such as *S. aureus*, *H. influenzae*, *Mycoplasma*, and *Legionella*; antibiotic coverage should also cover gram-negative organisms in older patients with comorbidities alongside smokers. For patients with chronic obstructive lung disease, gram-negative organisms, such as *Escherichia coli*, should be covered [66]. 

Beta-lactam antibiotics that target *S. pneumoniae*, such as amoxicillin combined with clavulanate for further coverage, are usually first-line treatments [66]. It is vital that we also choose antibiotics for treatment based on local community guidelines, patient profiles, comorbidities present, and potential for resistance. In patients who cannot tolerate amoxicillin, cefpodoxime can be administered; if all the abovementioned regimens cannot be tolerated, levofloxacin is recommended but discouraged in a healthy outpatient setting due to the risk of C. difficile infection. Lefamulin monotherapy has also been recommended, but caution should be exercised in patients who are pregnant or have arrhythmias or hepatic dysfunction [66]. The duration of antibiotic treatment for moderate pneumonia (pneumonia with additional symptoms including drowsiness, confusion, and worsening shortness of breath) is suggested at seven days. 

When treating a more severe and progressed version of this condition where patients typically do require hospitalization, a combination of beta-lactam antibiotics like cefotaxime and macrolides have shown to improve survival outcomes in not only ICU patients in sepsis (in this case, intravenous antibiotics need to be administered within one hour of symptom onset [67]) due to sCAP but also those presenting with non-pneumococcal sCAP [64,66-68], as compared to only beta-lactam monotherapy [69]. The use of levofloxacin monotherapy is also encouraged in this severe setting if the combination therapy cannot be tolerated [67]. In patients with sCAP requiring ICU admission, another recommended regime is combination therapy with either cefotaxime, ertapenem, or ampicillin-sulbactam combined with azithromycin or levofloxacin [67]. The duration of treatment here is guided by PCT (taken every 48 hours and compared to baseline at the time of diagnosis) levels, general patient stability, and response to treatment. Treatment regimens can be extended if the patient develops an extrapulmonary infection or has been infected with *Pseudomonas* [67].

Some systematic reviews [5,68] have demonstrated a fall in mortality of over 20% in hospitalized patients treated with macrolide antibiotics, such as azithromycin, compared with those that were on a non-macrolide regime. These drugs also provide coverage for aberrant pathogens such as *Mycoplasma*, *Chlamydophila*, and *Legionella* [64,68]. When treating Legionella, it is beneficial to combine macrolides with fluoroquinolones (to provide gram-negative cover) [64]. Regimes involving cephalosporins, such as ceftaroline [64], vancomycin, or linezolid [67], can be used to target MRSA and resistant variations of *S. pneumoniae* specifically due to the affinity of the drug to the pathogens’ penicillin-binding proteins, but this regime is currently not being considered for empirical therapy [64].

Adjunctive Glucocorticoids

The use of steroids alongside existing antibiotic regimens to treat sCAP helps to target the inflammatory processes of the disease, improve prognosis, and reduce all-cause mortality in patients [64,70,71]. Its use is specifically recommended in patients presenting with a systemic inflammatory response preceding shock, patients needing mechanical ventilation, or patients with respiratory failure (PaO2 to FiO2 ratio being less than 300) [67,72]. However, caution should be exercised in patients with poor blood glucose control and in those who are immunocompromised when using steroids, due to induction of subsequent hyperglycemia post-administration [64,67,70,71]. 

Influenza Therapy (Oseltamivir)

Several studies have provided evidence for the use of oseltamivir in treating severe pneumonia, especially within the first 48 hours from diagnosis [72,73]. Zanamivir has shown promise for the same purpose in immunocompromised patients [72].

New drugs for the treatment of sCAP

The emergence and widespread occurrence of antibiotic resistance poses a global public health concern, thus necessitating the development of novel antibacterial categories. Penicillin-resistant *S. pneumoniae* and ampicillin-resistant *H. influenzae* are ranked as priority pathogens on the World Health Organization’s roster for developing new antibiotics [9].

Lefamulin

Lefamulin was the first pleuromutilin antibiotic approved for intravenous and oral use in humans. Its unique mechanism involves binding to the peptidyl transferase site of bacterial ribosomes’ 50S subunit, consequently impeding the attachment of transfer RNA for peptide synthesis, thereby hindering protein production [10]. Lefamulin demonstrates efficacy against microorganisms that commonly cause community-acquired bacterial pneumonia, even against strains exhibiting resistance to other classes of antibiotics [10,74]. The drug reaches human tissues rapidly and reliably, with pulmonary epithelial lining fluid showing a mean 5.7-fold higher concentration than plasma [75]. In patients diagnosed with community-acquired bacterial pneumonia, a five-day oral course of lefamulin showed similar early clinical response to a seven-day oral treatment of moxifloxacin, observed at the 96-hour mark after initial dosage [74,75].

Delafloxacin

Oral and intravenous (IV) versions of delafloxacin, an anionic fluoroquinolone, are available. It has a different structure and charge profile than other quinolones, which results in an expanded spectrum of activity and side effects. In 2017, the US Food and Drug Administration granted approval for the use of delafloxacin in managing acute bacterial skin and skin structure infections (ABSSSI) [76]. Delafloxacin inhibits the activity of topoisomerase IV and DNA gyrase in both gram-positive and gram-negative bacteria. It has broad-spectrum efficacy against a wide array of bacteria, including methicillin-resistant *S. aureus*, and atypical and anaerobic strains.

The adverse effect profile of this drug is distinct from that of other fluoroquinolones, with the primary distinction being the lack of major central nervous system (CNS) events, phototoxicity, and corrected QT interval (QTc) prolongation [76]. In contrast to the dual treatment approach involving vancomycin and aztreonam, delafloxacin demonstrated non-inferiority while exhibiting a more favorable profile of adverse events [8]. Adults with community acquired bacterial pneumonia can be effectively treated with intravenous/oral delafloxacin monotherapy, which includes gram-positive, gram-negative, and atypical bacteria [76].

Omadacycline

Omadacycline is a novel, once-daily, oral, or intravenous antibiotic belonging to the aminomethylcycline class. As it reaches large quantities in pulmonary tissues, it is efficient against common pathogens that cause community-acquired bacterial pneumonia [9]. The observed safety profile of omadacycline matched the known safety profile of the tetracycline class. Adults with community-acquired bacterial pneumonia respond equally well to omadacycline and moxifloxacin treatments [9].

Prevention of sCAP

sCAP can be lethal, as up to 50% of patients die in patients who develop septic shock and, hence, require ICU admission [64]. Therefore, preventive measures are crucial. Primarily, behavioral risk factors, including smoking, alcohol intoxication, unhealthy diet, and lack of physical activity, need to be addressed. Pneumococcal vaccinations [64] and influenza vaccines have been readily available for many years, providing protection against two primary causes of pneumonia: *S. pneumoniae* and influenza virus. The pneumococcal vaccine is the most important vaccine given for the prevention of pneumonia, providing protection against the major culprit *S. pneumoniae* and reducing early mortality from invasive pneumococcal infection. Currently, the US has approved two variations of pneumococcal vaccines [77]:

(1) PPSV23 (pneumococcal polysaccharide vaccine): The vaccine protects against 23 types of pneumococci and is typically used in adults.

(2) PCV13 (pneumococcal conjugate vaccine): The vaccine was originally developed for infants and children.

These vaccinations are advised for all adults aged 65 years and above [78], smokers, immunocompromised patients [79], and patients with certain chronic illnesses.

The influenza vaccine reduces the risk of influenza-related pneumonia, which is a major complication of influenza infection. Annual influenza vaccination is recommended for individuals over the age of six months, healthcare workers, immunocompromised patients, and individuals with comorbidities [80]. Both active and passive smoking damage the lungs and reduce immunity to fight pneumonia. Smoking cessation is a crucial method to help prevent pneumonia and enhance general health. Nevertheless, maintaining hand hygiene, a healthy lifestyle, and control of underlying chronic conditions, such as asthma, diabetes, and congestive heart failure, can help prevent many health problems, including pneumonia.

Future research directions in pneumonia management

The management of sCAP continues to evolve as more research is being conducted on its prevalence. In addition to lefamulin, delafloxacin, and omadacycline, many other options have emerged to assist clinicians in dealing with the complexities of sCAP management.

While antibiotics continue to serve as a fundamental approach in pneumonia treatment, certain constraints are present, encompassing issues such as adverse effects, drug sensitivities, and antimicrobial resistance. Bacteriophage therapy has emerged as an adjunctive therapy, and case studies have shown promising results [1,81]. During the course of addressing *Pseudomonas* colonization, the case report by Maddocks et al. documented a successful de-escalation from an ICU unit to a high-dependency unit following seven-day administration of this intervention, with the absence of any untoward events both during and after treatment regimen [81]. While showing promise, substantial logistical challenges must be overcome before the widespread implementation of bacteriophage therapy can be implemented [1,82]. The susceptibility of an individual patient’s isolate to an array of bacteria-specific phages should be assessed. Typically, a combination of no fewer than three phages is required because of the tendency of resistance to manifest against singular phage agents [1]. Furthermore, it is important to note that the accessibility of phages and availability of susceptibility testing centers continue to be exceedingly restricted. Moreover, the optimal delivery method, specifically venous infusion, aerosolization, or instillation, remains uncertain and requires additional research [1].

Systemic corticosteroids exhibit favorable outcomes when administered to patients with sCAP. In a comprehensive meta-analysis conducted by Wu et al. [83], an examination of numerous randomized controlled trials [84-89] yielded the following findings. Firstly, a notably reduced mortality rate was observed among patients with sCAP compared with those who received placebo or conventional care in isolation. Secondly, a diminished mortality rate was evident in patients with sCAP who underwent corticosteroid treatment for a duration exceeding eight days in the absence of initial septic shock, with ICU admission and employment of hydrocortisone. Finally, patients who received this therapy had a shorter duration of stay in the ICU and hospital. However, the meta-analysis stressed the need for additional investigations, in light of inconclusive evidence, to substantiate the conclusions posited by the authors.

## Conclusions

In conclusion, this narrative review has delved into our current understanding of sCAP management, encapsulating the latest advancements in diagnostic and treatment modalities aimed at optimizing outcomes.

The epidemiology of sCAP underscores its significant impact on public health, particularly among older adults. The diverse etiology of sCAP emphasizes the role of bacterial and viral infections, with key pathogens such as *S. pneumoniae*, *Enterobacteriaceae*, *L. pneumophila*, and influenza virus identified as major contributors. In comparison to CURB, the PSI emerges as a reliable clinical tool for risk assessment and hospitalization decisions, complementing other diagnostic approaches. The multifaceted nature of sCAP diagnosis underscores the importance of a comprehensive, integrated approach for informed decision-making and optimized patient care.

Exploration of both non-pharmacological and pharmacological management strategies for sCAP has provided key insights. NIV, particularly helmet NIV, shows promise in reducing intubation needs and improving respiratory support during strenuous exertion. Immunoglobulins, such as IgM, and polyclonal antibodies, like IVIG and trimodulin, offer potential benefits in early sCAP stages. Emerging agents, like lefamulin, delafloxacin, and omadacycline, and antibiotic combinations of beta-lactam and macrolides have demonstrated substantial efficacy in migrating mortality risks. Adjunctive treatments, like steroids, show positive outcomes in clinical stability and hospital stays, although considerations for hyperglycemia must be taken into account. Preventative measures, including pneumococcal and influenza vaccinations, addressing behavioral risk factors and maintaining overall health continue to contribute to the reduction of sCAP incidence. Future research directions involve exploring novel treatments like bacteriophage therapy and further investigating the efficacy and optimal administration of systemic corticosteroids. Despite these advancements, the complexities of sCAP management necessitate ongoing research and clinical exploration to enhance patient outcomes.
